# An alternative approach to study irrotational periodic gravity water waves

**DOI:** 10.1007/s00033-021-01578-8

**Published:** 2021-07-05

**Authors:** Biswajit Basu, Calin I. Martin

**Affiliations:** 1 School of Engineering, Trinity College Dublin, Dublin 2, Ireland; 2 Faculty of Mathematics, University of Vienna, Oskar-Morgenstern-Platz 1, 1090 Vienna, Austria

**Keywords:** Flow force, Free surface flows, Stagnation points, Overhanging profiles, Local bifurcation, 35Q31, 35Q35, 76B15.

## Abstract

We are concerned here with an analysis of the nonlinear irrotational gravity water wave problem with a free surface over a water flow bounded below by a flat bed. We employ a new formulation involving an expression (called flow force) which contains pressure terms, thus having the potential to handle intricate surface dynamic boundary conditions. The proposed formulation neither requires the graph assumption of the free surface nor does require the absence of stagnation points. By way of this alternative approach we prove the existence of a local curve of solutions to the water wave problem with fixed flow force and more relaxed assumptions.

## Introduction

The paper by Benjamin and Lighthill [3], back in 1958, highlighted a quantity called *flow force*–defined as the rate of momentum of fluid flow (corrected for pressure and normalized with respect to density)–and emphasized its role as the driver of the flow. Research on flow force was greatly enhanced by the study of Keady and Norbury [23]. Recently, a novel flow force formulation of the free surface irrotational nonlinear water wave problem has been proposed by Basu [1] by way of exploiting the invariance property of the flow force on the fluid’s bed and on the free surface. This formulation was further extended by Basu and Martin [2] to bear relevance to the case of capillary-gravity water waves. For this, a suitable modification of the definition of the flow force was employed. Flow force has also been used to uniquely parameterize the subset of waves with crests located on a fixed vertical line and to verify the Benjamin-Lighthill conjecture with values of Bernoulli’s constant close to a critical value by Kozlov et al. [24].

While the research on periodic traveling water waves was initiated in 1847 by Stokes [36], a truly remarkable development occurred in the beginning of the 20th century with the works by Nekrasov [34], Levi–Civita [26], Struik [37] in 1920s and continued by Krasovskii [25], Keady and Norbury [23], McLeod [33] and Toland [39]. However, thorough analyses of water flows with vorticity emerged only (relatively) recently: indeed, the existence of large-amplitude water waves with vorticity was proved rigorously by Constantin and Strauss [12] in 2004. The latter study had further surged additional research in the area as can be seen by consulting the following (selective) list [4–9, 11, 13, 14, 16, 19, 20, 42, 43]. Although fluid flows display to a large extent vorticity, irrotational flows are worthy of attention not only as idealized mathematical models but also as physical occurrences, since it is known that zero vorticity models uniform currents. Moreover, irrotational flows constitute, at times, valid approximations for a number of physical scenarios that come close to lacking vorticity.

Here we devote our attention to the existence of solutions in the irrotational gravity water wave problem. Instrumental in achieving our goal is a flow force reformulation of the free surface, periodic, irrotational, gravity water wave problem. Recent flow force reformulations of the irrotational water wave problem were performed recently in the capillary-gravity case ( [2]) and in the gravity scenario ( [1]). The inclusion of vorticity (in the flow force scenario) still remains to be considered. For a selection of studies presenting exact solutions for the rotational water wave problem with surface tension we refer the reader to [21, 22, 27–32, 41].

In the previous studies, the height function formulation has been one of the popular approaches for dealing with the water wave problem. The formulation assumes that the mass flux of the flow is fixed throughout the bifurcation curve. Here, we take a different route and, instead of fixing the mass flux, we search for water flows with fixed flow force. We then use an approach devised by Constantin & Varvaruca [16] **which, by means of the Hilbert transform and of the Dirichlet–Neumann operator, is able to recast the water wave problem as single quasilinear equation for the free surface.** Subsequently we avail of bifurcation-type results and prove the existence of a solution curve consisting of exact periodic solutions to the irrotational gravity water wave problem in water of finite depth by a method different from the one in [1]. More precisely, while the height function approach (utilized in [1]) **can only handle flows whose free surface is a graph and which lack stagnation points, the method we use in this paper is not bound to the previous two assumptions. Therefore, the procedure that we undertake here enables us to cover overhanging profiles and opens up new perspectives for dealing with more complex scenarios like the presence of vorticity** [16, 27] known to be a trigger of critical layers and stagnation points [10, 35] and a facilitator of overhanging profiles [15, 18, 38].

The layout of the paper is as follows: Sect. 2 is used to introduce the water wave problem in terms of the velocity formulation and then by availing of the stream function. We then introduce the reformulation of the water wave problem by means of the flow force function. Essential properties of the flow force function are also presented. We conclude Sect. 2 by showing the equivalence of the water wave problem with a single quasilinear equation satisfied by the free surface. Instrumental in proving the previous equivalence are the *Hilbert transform* and the *Dirichlet–Neumann operator* whose properties are also recalled. The main result showing the existence of a local curve of solutions to the water wave problem in the context of a fixed flow force is presented in Sect. 3. The last section of the paper lists several properties of the Hilbert transform and of the Dirichlet–Neumann operator used throughout the paper.

## The water wave problem and several reformulations

**The fluid domain and the water wave problem**

We recall here the problem of spatially periodic travelling irrotational gravity water waves in a flow of finite depth. It is a free-boundary problem which consists of finding

- a domain $\Omega$ in the (*X*, *Y*)-plane which is bounded below by the flat bed
  $$\begin{aligned} {\mathscr {B}} = \{(X,0); X \in {\mathbb {R}}\}, \end{aligned}$$
  and above by an a priori unknown curve
  2.1 $$\begin{aligned} {\mathscr {S}} = \{(u(s),v(s)); s\in {\mathbb {R}}\},\,\, \end{aligned}$$
  satisfying
  2.2 $$\begin{aligned} (u_s(s))^2+(v_s(s))^2>0\,\,\mathrm{for}\,\,\mathrm{all}\,\,s\in {\mathbb {R}},\,t\ge 0 \end{aligned}$$
  and
  2.3 $$\begin{aligned} u(t,s+L)=u(t,s)+L\quad v(t,s+L)=v(t,s)\,\,\mathrm{for}\,\,\mathrm{all}\,\,s\in {\mathbb {R}},\,t\ge 0 \end{aligned}$$
  where $L>0$ is a constant that denotes the period of the motion;
- a pair of periodic functions $(u_1,u_2)$, representing the velocity field and a periodic function *P*, denoting the pressure, required to satisfy within the domain $\Omega$ the conservation of momentum equations
  2.4 $$\begin{aligned} \begin{aligned} (u_1-c)u_{1X}+u_2 u_{1Y}&=-P_X,\\ (u_1-c)u_{2X}+u_2 u_{2Y}&=-P_Y-g, \end{aligned} \end{aligned}$$
  (with *c* being the surface wave speed) and the equation of mass conservation
  2.5 $$\begin{aligned} u_{1X}+u_{2Y}=0, \end{aligned}$$
  together with suitable boundary conditions of kinematic and dynamic type, which we now specify. Indeed, stating in mathematical terms that ${\mathscr {S}}$ represents the surface boundary of the flow amounts to requiring for all $s\in {\mathbb {R}}$
  2.6 $$\begin{aligned} (u_1(u(s),v(s))-c){\mathscr {E}}_X(u(s),v(s))+u_2(u(s),v(s)) {\mathscr {E}}_Y(u(s),v(s))=0, \end{aligned}$$
  where ${\mathscr {E}}(X,Y)=0$ is an implicit equation of the free surface for a parameterization for which *u* and *v* are time independent in the moving frame. Equation (2.6) is the kinematic boundary condition on the surface and ensures that ${\mathscr {S}}$ is an impenetrable boundary. Analogously, to render the bottom the impermeability status, we require no motion in the vertical direction, that is
  2.7 $$\begin{aligned} u_2(X,0)=0\quad \mathrm{for}\,\,\mathrm{all}\,\,X\in {\mathbb {R}}. \end{aligned}$$
  Moreover, the dynamic boundary condition
  2.8 $$\begin{aligned} P= P_{atm}\,\,\mathrm{on}\,\,{\mathscr {S}}, \end{aligned}$$
  where $P_{atm}$ is the constant atmospheric pressure and decouples the motion of the air above the free surface from the motion of the water. While, certainly, flows exhibiting vorticity are most interesting, we are confining ourselves to irrotational flows to which the method of work in this paper seems to apply more directly. The irrotationality of the flow is expressed analytically by the condition
  2.9 $$\begin{aligned} u_{1_Y}-u_{2_X}\equiv 0. \end{aligned}$$

In the following we introduce the stream function $(X,Y)\rightarrow \psi (X,Y)$, defined (up to an additive constant) by the relations

2.10 $$\begin{aligned} \psi _X=-u_2, \quad \psi _Y=u_1-c \ , \end{aligned}$$

and which allows for a simpler formulation of the water wave problem by reducing the number of unknowns.

Relations(2.10) and (2.9) imply that $\psi$ is a harmonic function.

Valuable insights into how the flow flows is delivered by the mass flux (relative to the uniform flow *c*) which is defined as

2.11 $$\begin{aligned} p_0=\mathop {\mathrm {\int }}\limits _{0}^{\eta (X)} (u_1(X,Y)-c) dY. \end{aligned}$$

From the equation of mass conservation (2.5) and from (2.6) we deduce that $p_0$ is, in fact, a constant.

Another invariant quantity of the flow appears in connection with Bernoulli’s law which is derived with the help of the Euler‘s equations and states that

2.12 $$\begin{aligned} E := \frac{(c-u_{1})^2+u_2^2}{2}+gY+P \end{aligned}$$

is a constant within the fluid. Consequently, the dynamic boundary condition at the surface can be restated as

2.13 $$\begin{aligned} \psi ^2_X+\psi _Y^2+2gY =Q~~\text {on}~~{\mathscr {S}} \end{aligned}$$

where $Q=2(E-P_{atm})$ is a constant (called the hydraulic head) for any given flow. The previous considerations pertaining to the stream function allow us to reformulate the free surface irrotational gravity water wave problem as the system [12]

2.14 $$\begin{aligned} \begin{aligned}&\bigtriangleup \psi = 0 ~~\text {in}~~\Omega ,\\&\quad |\nabla \psi |^2+2g Y =Q~~\text {on}~~{\mathscr {S}},\\&\quad \psi =0~~\text {on}~~{\mathscr {S}},\\&\quad \psi =-p_0~~\text {on}~~Y=0, \end{aligned} \end{aligned}$$

which is to be solved for functions which are *L*-periodic in the $X-$variable within the fluid domain $\Omega$.

**Reformulation of the water wave problem via the flow force function**

The governing equations introduced at the beginning of this section will be written in an equivalent manner using the flow force function, cf. [3, 23, 24]. We recall that the flow force function is given by the expression

2.15 $$\begin{aligned} S(X,Y) = \mathop {\mathrm {\int }}\limits _{0}^Y [p(X,r) + ( u_{1}(X,r) - c)^2] \mathrm{d}r \ \ \end{aligned}$$

where $p(X,Y)=P(X,Y)-P_{atm}$. In the following lemma we prove an important property of *S* which makes it suitable in the reformulation of the water wave problem.

### Lemma 1

The flow force function *S* is constant on the two boundaries of the fluid domain.

### Proof

Indeed, it is immediate from (2.15) that

$$\begin{aligned} \begin{array}{ll} S = 0 &{} \text{ on } \text{ the } \text{ bottom } {\mathscr {B}}. \\ \end{array} \end{aligned}$$

We will show in the following that *S* is constant on the free surface. To this end we note that the gradient of *S* satisfies

2.16 $$\begin{aligned} S_X=-(u_1-c)u_2,\qquad S_Y=p(X,Y)+(u_1(X,Y)-c)^2, \end{aligned}$$

where the first equation above follows from the equality

2.17 $$\begin{aligned} P_X + 2(u_{1}-c) {u_{1}}_X = -(u_{1}-c) {u_{2}}_Y - u_{2}{u_{1}}_Y = -\{(u_{1}- c)u_{2}\}_Y , \end{aligned}$$

which is a consequence of the Euler and mass conservation equation. Moreover, for the parameterization $\{(u(s),v(s)):s\in {\mathbb {R}}\}$ of the free surface ${\mathscr {S}}$ it holds that ${\mathscr {E}}(u(s),v(s))=0$ for all $s\in {\mathbb {R}}$, which implies that

2.18 $$\begin{aligned} {\mathscr {E}}_X(u(s),v(s))u_s+{\mathscr {E}}_Y(u(s),v(s))v_s=0\,\,\mathrm{for}\,\,\mathrm{all}\,\,s\in {\mathbb {R}}. \end{aligned}$$

From the latter equality and using also surface kinematic condition (2.6) we infer that $(u_1(u(s),v(s))-c,u_2(u(s),v(s))$ and $(u_s,v_s)$ are parallel for all $s\in {\mathbb {R}}$, that is there is $s\rightarrow \alpha (s)$ such that

$$\begin{aligned} (u_s,v_s)=\alpha (s)\big (u_1(u(s),v(s))-c,u_2(u(s),v(s)\big )\,\,\mathrm{for}\,\,\mathrm{all}\,\,s\in {\mathbb {R}}. \end{aligned}$$

Therefore, setting $S_0(X):=S(u(s),v(s))$ we find that

2.19 $$\begin{aligned} \begin{aligned} \frac{dS_0}{ds}&= S_X(u(s),v(s))u_s+S_Y(u(s),v(s))v_s\\&= \alpha (s)\big (S_X(u(s),v(s)) (u_1-c) +S_Y(u(s),v(s)) u_2\big )\\&= \alpha (s)\big (-u_2(u_1-c)^2+(p(u(s),v(s))+(u_1-c)^2)u_2\big )\\&=0 \end{aligned} \end{aligned}$$

where, to obtain the last equalities above, we have also used the dynamic and surface kinematic conditions and that $p(X,Y)=P(X,Y)-P_{atm}$. Thus, $S_0$ is, in fact, a constant. $\square$

Differentiating now with respect to *X* and *Y* in (2.16) we obtain

2.20 $$\begin{aligned} S_{XX} + S_{YY} =- g - 2(c-u_{1}) ({u_1}_Y - {u_{2}}_X) \end{aligned}$$

which, in the absence of vorticity, leads to

2.21 $$\begin{aligned} \Delta S = -g \end{aligned}$$

with $S = 0$ on the flat bed ${\mathscr {B}}$ and $S = S_0$ on the free surface ${\mathscr {S}}$. The nonlinear dynamic boundary condition on the free surface may be expressed as

2.22 $$\begin{aligned} \left( \frac{S_X ^2}{S_Y } + S_Y \right) \Big |_{{\mathscr {S}}} +2 g v(s) = Q. \end{aligned}$$

The previous considerations show that water wave problem (2.14) can be written in terms of the flow force function *S* as

2.23 $$\begin{aligned} \begin{aligned}&\Delta S = -g,\\&\quad S=S_0\,\,\mathrm{on}\,\,{\mathscr {S}},\\&\quad S=0\,\,\mathrm{on}\,\,{\mathscr {B}},\\&\quad \left( \frac{S_X ^2}{S_Y } + S_Y \right) \Big |_{{\mathscr {S}}} +2 g v(s)= Q. \end{aligned} \end{aligned}$$

**The water wave problem as a quasilinear equation for the free surface**

We will aim to further reformulate problem (2.23) as a single equation for the free surface ${\mathscr {S}}$. To this end, we need a few notations and some preliminary results. For the proof of these results we refer the reader to [16].

### Definition 2

For $p\in {\mathbb {N}}$ and $\alpha \in (0,1)$ we denote by $C^{p,\alpha }$ the space of Hölder continuous functions. The space of functions of class $C^{p,\alpha }$ over any compact subset of their domain of definition will be denoted with $C_{\text {loc}}^{p,\alpha }$. Moreover, by $C_{2\pi }^{p,\alpha }$ we denote the space of functions of one real variable which are $2\pi$ periodic and of class $C_{\text {loc}}^{p,\alpha }$ in ${\mathbb {R}}$, while by $C_{2\pi , o}^{p,\alpha }$ we denote the functions that are in $C_{2\pi }^{p,\alpha }$ and have zero mean over one period. Finally, by $L_{2\pi }^{2}$ we denote the space of $2\pi$-periodic locally square integrable functions of one real variable. By $L_{2\pi ,\text {o}}^{2}$ we denote the subspace of $L_{2\pi }^{2}$ whose elements have zero mean over one period.

The next definition introduces the *Dirichlet–Neumann operator* associated with a horizontal strip in the plane

### Definition 3

For $d>0$ we set

$$\begin{aligned} {\mathscr {R}}_{d}:=\{(x,y)\in {\mathbb {R}}^{2}:-d<y<0\}. \end{aligned}$$

Given $w\in C_{2\pi }^{p,\alpha }$ we let $W\in C^{p,\alpha }(\overline{{\mathscr {R}}}_{d})$ be the unique solution of

2.24 $$\begin{aligned} \begin{array}{rcl} \Delta W &{} = &{} 0\,\,\text {in}\,\,{\mathscr {R}}_{d},\\ W(x,-d) &{} = &{} 0,\,\, x\in {\mathbb {R}},\\ W(x,0) &{} = &{} w(x),\,\,x\in {\mathbb {R}}. \end{array} \end{aligned}$$

The function $(x,y)\rightarrow W(x,y)$ is $2\pi$-periodic in *x* throughout ${\mathscr {R}}_{d}$. For $p\in {\mathbb {Z}},p\ge 1$, and $\alpha \in (0,1)$ we define the *periodic Dirichlet–Neumann operator for a strip* ${\mathscr {G}}_{d}$ by

$$\begin{aligned} {\mathscr {G}}_{d}(w)(x):=W_{y}(x,0),\,\,x\in {\mathbb {R}}. \end{aligned}$$

In addition to the Dirichlet–Neumann operator we will need the *periodic Hilbert transform* for the strip ${\mathscr {R}}_d$. More precisely, recalling the function *W* from Definition 3 and setting *Z* to be the unique (up to a constant) harmonic function such that $Z+iW$ is holomorphic in ${\mathscr {R}}_d$, we define for $w\in C_{2\pi , o}^{p,\alpha }$

$$\begin{aligned} {\mathscr {C}}_d(w)(x):=Z(x,0)\,\,\mathrm{for}\,\,x\in {\mathbb {R}}. \end{aligned}$$

The map ${\mathscr {C}}_d$ is called the *periodic Hilbert transform* for the strip ${\mathscr {R}}_d$. Some useful properties of the Dirichlet–Neumann operator and of the Hilbert transform are listed in Sect. 3.

The following two definitions specify the type of solutions we will be proving to exist for free boundary value problem (2.23).

### Definition 4

We say that a solution $(\Omega , S)$ of water wave problem (2.23) is of class $C^{1,\alpha }$ if the free surface belongs to $C^{1,\alpha }_{2\pi }$ and $S\in C^{\infty }(\Omega )\cap C^{1,\alpha }({\overline{\Omega }})$.

### Definition 5

- We say that $\Omega \subset {\mathbb {R}}^{2}$ is an *L-periodic strip like domain* if it is contained in the upper half (*X*, *Y*)-plane and if its boundary consists of the real axis ${\mathscr {B}}$ and a parametric curve ${\mathscr {S}}$ defined by (2.1) which is *L*-periodic.
- For any such domain, the *conformal mean depth* is defined to be the unique positive number *h* such that there exists an onto conformal mapping ${\tilde{U}}+i{\tilde{V}}:{\mathscr {R}}_{h}\rightarrow \Omega$ which admits an extension between the closures of these domains, with onto mappings
  $$\begin{aligned} \{(x,0):x\in {\mathbb {R}}\}\rightarrow {\mathscr {S}}, \end{aligned}$$
  and
  $$\begin{aligned} \{(x,-h):x\in {\mathbb {R}}\}\rightarrow {\mathscr {B}}, \end{aligned}$$
  and such that
  2.25 $$\begin{aligned} \begin{array}{lll} {\tilde{U}}(x+L,y) &{} = &{}{\tilde{U}}(x,y)+L\\ {\tilde{V}}(x+L,y) &{} = &{}{\tilde{V}}(x,y) \end{array}\,\,\,\,\mathrm{for}\,\,\mathrm{all}\,\, (x,y)\in {\mathscr {R}}_{h}. \end{aligned}$$

The existence and uniqueness of such an *h* was proved by Constantin & Varvaruca in Appendix A of [16].

We are now ready to state the main result of the current section, namely the reformulation of the water wave problem as a quasilinear equation for a periodic function (representing the elevation of the free surface) of one variable in a fixed domain.

### Theorem 6

If $(\Omega , S)$ of class $C^{1,\alpha }$ is a solution of (2.23) then there exist a positive number h, a positive function $v\in C^{1,\alpha }_{2\pi }$, with $[v]=h$, and a constant a $\in {\mathbb {R}}$ such that

2.26 $$\begin{aligned} \frac{Bv^{\prime 2}}{({\mathscr {G}}_{kh}(v)^2+v'^2){\mathscr {G}}_{kh}(v) } + \frac{ B{\mathscr {G}}_{kh}(v)}{{\mathscr {G}}_{kh}(v)^2+v'^2} =Q -2gv \end{aligned}$$

where $k = \frac{2\pi }{L}$ and *B* is an operator defined through

2.27 $$\begin{aligned} C^{1,\alpha }_{2\pi } \ni v\rightarrow Bv= \frac{S_0}{kh}+g({\mathscr {G}}_{kh}(\frac{v^2}{2})-v{\mathscr {G}}_{kh}(v)). \end{aligned}$$

Moreover, the surface boundary is given as

2.28 $$\begin{aligned} \begin{aligned}&{\mathscr {S}} = \left\{ \left( a + \frac{x}{k} + {\mathscr {C}}_{kh}(v - h)(x),v(x) \right) :x\in {\mathbb {R}}\right\} , \end{aligned} \end{aligned}$$

whereby, the mapping

$$\begin{aligned} x \rightarrow \left( \frac{x}{k} + {\mathscr {C}}_{kh}(v - h)(x), v(x) \right) \end{aligned}$$

is injective on ${\mathbb {R}}$ and

2.29 $$\begin{aligned} v'(x)^2 + {\mathscr {G}}_{kh}(v)(x)^2 \ne 0\ for\ all\ x\ \in \ {\mathbb {R}} . \end{aligned}$$

Conversely, let $h>0$ and $v \in C^{1,\alpha }_{2\pi }$ be such that (2.26) holds. Assume also that ${\mathscr {S}}$ is defined by (2.28), let $\Omega$ be the domain whose boundary consists of ${\mathscr {S}}$ and of the real axis ${\mathscr {B}}$ and let $a\in {\mathbb {R}}$ be arbitrary. Then there exists a function *S* in $\Omega$ such that $(\Omega ,S)$ is a solution of (2.23) of class $C^{1,\alpha }$.

### Proof

We follow largely the line of work from [16]. Let us assume first that $(\Omega ,S)$ is a solution of class $C^{1,\alpha }$ of (2.23). We set *h* to be the conformal mean depth of $\Omega$ and denote by ${\tilde{U}}+i{\tilde{V}}$ the conformal mapping corresponding to $\Omega$, as in Definition 5.

We adapt now the conformal mapping technique to our purposes and bring in the wave number *k* by setting for all $(x,y)\in {\mathscr {R}}_{kh}$

2.30 $$\begin{aligned} U(x,y): = {\tilde{U}} \left( \frac{x}{k},\frac{y}{k}\right) \quad \mathrm{and}\quad V(x,y): = {\tilde{V}} \left( \frac{x}{k},\frac{y}{k}\right) . \end{aligned}$$

The latter assignment defines a mapping $U+iV:{\mathscr {R}}_{kh} \rightarrow \Omega$. Conducting an analysis as in the proof of Theorem 2.2 in [16] we see that $U,V \in C^{1,\alpha }(\overline{{\mathscr {R}}_{kh}})$ and that $U + iV$ is a conformal mapping from ${\mathscr {R}}_{kh}$ onto $\Omega$ which extends homeomorphically to the closures of these domains, with onto mappings

$$\begin{aligned} \{(x,0):x \in {\mathbb {R}}\} \rightarrow {\mathscr {S}}\qquad \mathrm{and}\qquad \{(x,-kh): x \in {\mathbb {R}}\} \rightarrow {\mathscr {B}}. \end{aligned}$$

Also, nondegeneracy condition (2.2) becomes

$$\begin{aligned} U_x^2(x,0) + V_x^2(x,0) \ne 0 \,\,\mathrm{for}\,\, \mathrm{all}\,\, x \in {\mathbb {R}}. \end{aligned}$$

For the purpose of writing the surface boundary ${\mathscr {S}}$ with the help of the new coordinates we put for all $x\in {\mathbb {R}}$

$$\begin{aligned} v(x): = V(x,0) \quad \mathrm{and} \quad u(x): = U(x,0) . \end{aligned}$$

Therefore, it holds that

$$\begin{aligned} u = {\mathscr {C}}_{kh}(v) \end{aligned}$$

and

2.31 $$\begin{aligned} u' = {\mathscr {G}}_{kh}(v) \ and \ u'' = {\mathscr {G}}_{kh}(v'). \end{aligned}$$

Analogously as in [16] one can show that $v\in C_{2\pi }^{1,\alpha }$ and

$$\begin{aligned}{}[v]=h, \ \ \ \ \end{aligned}$$

$$\begin{aligned} v(x) > 0 \,\,\mathrm{for}\,\, \mathrm{all}\,\, x\in {\mathbb {R}}, \end{aligned}$$

$$\begin{aligned} \mathrm{the}\,\,\mathrm{map}\,\, x\rightarrow \left( \frac{x}{k} + {\mathscr {C}}_{kh}(v - h)(x), v(x) \right) \,\,\mathrm{is}\,\,\mathrm{injective}\,\, \mathrm{on}\,\, {\mathbb {R}}, \end{aligned}$$

2.32 $$\begin{aligned} {\mathscr {S}}=\left\{ \left( a + \frac{x}{k} + {\mathscr {C}}_{kh}(v - h)(x),v(x) \right) : x \in {\mathbb {R}} \right\} , \end{aligned}$$

for some $a \in {\mathbb {R}}$, which arises in formula (2.32) when noticing that problem (2.23) is invariant under horizontal translations. The assertion

$$\begin{aligned} v'(x)^2 + {\mathscr {G}}_{kh}(v)(x)^2 \ne 0\,\,\mathrm{for}\,\,\mathrm{all}\,\, x\in {\mathbb {R}} \end{aligned}$$

is true by (2.31) and the Cauchy–Riemann equations.

Now let $\xi : {\mathscr {R}}_{kh} \rightarrow {\mathbb {R}}$ be defined by

2.33 $$\begin{aligned} \xi (x,y) = S(U(x,y),V(x,y)),\ (x,y) \in {\mathscr {R}}_{kh}. \end{aligned}$$

The harmonicity in $\Omega$ of the function $(X,Y)\rightarrow S(X,Y)+\frac{g}{2}Y^2$ and the invariance of harmonic functions under conformal mappings imply that

$$\begin{aligned} \xi + \frac{g}{2}V^2 \ \text {is harmonic in} \ {\mathscr {R}}_{kh}. \end{aligned}$$

Availing of the Cauchy–Riemann equations and of the chain rule supplies us with the formula

2.34 $$\begin{aligned} \begin{pmatrix} S_X\\ S_Y \end{pmatrix}\Big |_{(U(x,y),V(x,y))} = \frac{1}{v'^2+{\mathscr {G}}_{kh}(v)^2} \begin{pmatrix} V_y &{} -V_x \\ V_x &{} V_y \end{pmatrix} \begin{pmatrix} \xi _x \\ \xi _y \end{pmatrix} \ in \ \overline{{\mathscr {R}}_{kh}}. \end{aligned}$$

Define $$\zeta :{\mathscr {R}}_{kh}\rightarrow {\mathbb {R}}$$ through

2.35 $$\begin{aligned} \zeta (x,y)= \xi (x,y)+\frac{g}{2}V^2(x,y). \end{aligned}$$

Using the boundary conditions we obtain that $\zeta$ verifies the system

2.36 $$\begin{aligned} \begin{aligned} \bigtriangleup \zeta&= 0 \ in \ {\mathscr {R}}_{kh}\\ \zeta (x,-kh)&=0 \,\,\mathrm{for}\,\,\mathrm{all}\,\, x\in {\mathbb {R}}\\ \zeta (x,0)&= S_0 +\frac{g}{2}v^2\,\,\mathrm{for}\,\,\mathrm{all}\,\, x\in {\mathbb {R}}, \end{aligned} \end{aligned}$$

from which we infer that

$$\begin{aligned} \zeta _y(x,0)={\mathscr {G}}_{kh}\left( S_0 + \frac{g}{2}v^2\right) . \end{aligned}$$

We clearly have $\xi (x,0)=S(U(x,0),V(x,0))=S_0$ and therefore, $\xi _x(x,0)=0$ for all $x\in {\mathbb {R}}$. Moreover,

2.37 $$\begin{aligned} \xi _y (x,0) = \zeta _y(x,0) - g(VV_y)(x,0) = \frac{S_0}{kh}+g({\mathscr {G}}_{kh}(\frac{v^2}{2})-v{\mathscr {G}}_{kh}(v))=:B. \end{aligned}$$

Availing now of (2.34) we have that

2.38 $$\begin{aligned} \begin{aligned} S_X\Big |_{(U(x,0),V(x,0))}&=-\frac{Bv'}{v'^2+{\mathscr {G}}_{kh}(v)^2},\\ S_Y\Big |_{(U(x,0),V(x,0))}&=\frac{{\mathscr {G}}_{kh}(v) B}{v'^2+{\mathscr {G}}_{kh}(v)^2}. \end{aligned} \end{aligned}$$

Hence, the last equation of (2.23) can be rewritten as

$$\begin{aligned} \frac{Bv'^2}{({\mathscr {G}}_{kh}(v)^2+v'^2){\mathscr {G}}_{kh}(v) } + \frac{B{\mathscr {G}}_{kh}(v)}{{\mathscr {G}}_{kh}(v)^2+v'^2} =Q -2gv, \end{aligned}$$

that is, (2.26) holds true.

In order to prove the sufficiency part we suppose that the positive number *h* and the function $v \in C_{2\pi }^{1,\alpha }$ satisfy (2.26). Let $V:{\mathscr {R}}_{kh}\rightarrow \Omega$ which satisfies

2.39 $$\begin{aligned} \begin{aligned} \Delta V&=0\,\,\mathrm{in}\,\, {\mathscr {R}}_{kh} \\ V(x,-kh)&=0\,\, \mathrm{all}\,\, x \in {\mathbb {R}}\\ V(x,0)&= v(x) \,\,\mathrm{for}\,\, \mathrm{all}\,\, x \in {\mathbb {R}}, \end{aligned} \end{aligned}$$

and let $U:{\mathscr {R}}_{kh} \rightarrow \Omega$ be such that $U+iV$ is holomorphic. An application of Lemma 2.1 from [16] yields that $U+iV \ \in \ C^{2,\alpha }( \overline{{\mathscr {R}}_{kh}})$. From $[v]=h$ we obtain

2.40 $$\begin{aligned} \left\{ \begin{aligned}&U(x+2\pi ,y)=U(x,y)+\frac{2\pi }{k}\\&V(x+2\pi ,y)=V(x,y) \end{aligned}\,\,\,\, \mathrm{for}\,\,(x,y) \ \in \ {\mathscr {R}}_{kh}. \right. \end{aligned}$$

The injectivity of the mapping $x \rightarrow \big (\frac{x}{k}+{\mathscr {C}}_{kh}(v - h)(x), v(x) \big )$ yields that curve (2.28) is nonself-intersecting, while from $v(x) > 0$ we have that (2.28) is contained in the upper half-plane. If $\Omega$ denotes the domain whose boundary consists of ${\mathscr {S}}$ and ${\mathscr {B}}$, it follows from Theorem 3.4 in [40] that $U+iV$ is a conformal mapping from ${\mathscr {R}}_{kh}$ onto $\Omega$, which extends to a homeomorphism between the closures of these domains, with onto mappings

$$\begin{aligned} \{(x,0):x \ \in \ {\mathbb {R}}\} \rightarrow {\mathscr {S}}\,\,\mathrm{and}\,\, \{(x,-kh):x \ \in \ {\mathbb {R}}\} \rightarrow {\mathscr {B}}. \end{aligned}$$

The latter assertion and (2.40) show that $\Omega$ is an L-periodic strip-like domain, with $L = 2\pi /k$. Moreover, the conformal mean depth of $\Omega$ is *h* as it can be derived from the properties of the mapping ${\overline{U}}+i{\overline{V}}:{\mathscr {R}}_h \rightarrow \Omega$, where ${\overline{U}}$,${\overline{V}}$ are given by (2.30). Let $\zeta$ be defined as the unique solution of (2.36). Then $\zeta \in C^{1,\alpha }(\overline{{\mathscr {R}}_{kh}}) \cap C^\infty ({\mathscr {R}}_{kh})$. Now, let $\xi$ be defined by (2.35) and *S* by (2.33). We obtain that *S* satisfies (2.23) along with the boundary conditions. From (2.26) we also have that *S* satisfies the last equation from (2.23).

$$\square $$

## Existence of exact solutions

We will be concerned here with an analysis of equation (2.26) by means of local bifurcation techniques, cf. [17]. To begin with, prompted by relation $[v]=h$, we set

3.1 $$\begin{aligned} v=w+h. \end{aligned}$$

Equation (3.1) implies immediately that $[w]=0$. We then use [16] to find that

$$\begin{aligned} {\mathscr {G}}_{kh}(v)={\mathscr {G}}_{kh}(w+h)={\mathscr {G}}_{kh}(w) + {\mathscr {G}}_{kh}(h)=\frac{1}{k}+{\mathscr {C}}_{kh}(w') \end{aligned}$$

and

$$\begin{aligned} {\mathscr {G}}_{kh}(v')={\mathscr {G}}_{kh}(w')=\frac{[w']}{kh}+{\mathscr {C}}_{kh}(w'')={\mathscr {C}}_{kh}(w''), \end{aligned}$$

since *w* is periodic. Therefore, we can rewrite (2.26) as

3.2 $$\begin{aligned} \frac{ B}{\left( w^{'2}+\left( \frac{1}{k}+{\mathscr {C}}_{kh}(w') \right) ^2\right) } \left( \frac{w^{\prime 2}}{ \frac{1}{k}+{\mathscr {C}}_{kh}(w') }+\frac{1}{k}+{\mathscr {C}}_{kh}(w') \right) =Q -2gh-2gw \end{aligned}$$

with

3.3 $$\begin{aligned} B = \frac{S_0 }{kh} + g\left( \frac{[w^2]}{2kh} - \frac{w}{k} - \frac{h}{2k} + {\mathscr {C}}_{kh}(w w') - w {\mathscr {C}}_{kh}(w') \right) . \end{aligned}$$

Moreover, the map $x\rightarrow \left( \frac{x}{k}+{\mathscr {C}}_{kh}(w)(x),w(x)+h \right)$ is injective on ${\mathbb {R}}$ and $w'(x)^2+\left( \frac{1}{k}+{\mathscr {C}}_{kh}(w')(x)\right) ^2 \ne 0$ for all $x\in {\mathbb {R}}$

We will regard $$S_0$$ and *Q* as parameters and will prove the existence of solutions $$w\ \in \ C_{2\pi }^{1,\alpha }$$ to equation (3.2) for all $$ k>0$$ and $$h>0$$ fixed. We observe that $$w=0\ \in \ C_{2\pi }^{1,\alpha }$$ is a solution of (3.2) if and only if

3.4 $$\begin{aligned} Q = 2gh+\left( \frac{S_0}{h}- \frac{gh}{2}\right) . \end{aligned}$$

The latter equality suggests setting

3.5 $$\begin{aligned} \lambda = \frac{S_0}{h} - \frac{gh}{2}, \end{aligned}$$

3.6 $$\begin{aligned} \mu = Q - 2gh - \left( \frac{S_0}{h} - \frac{gh}{2} \right) . \end{aligned}$$

For the further developments it is important to note that the mapping $(S_0,Q)\rightarrow (\lambda ,\mu ))$ is a bijection from ${\mathbb {R}}^2$ onto itself. With notation (3.4)-(3.6) we have

3.7 $$\begin{aligned} \begin{aligned}&B=\frac{\lambda }{k} + g\left( \frac{[w^2]}{2kh} - \frac{w}{k} + {\mathscr {C}}_{kh}(w w') - w {\mathscr {C}}_{kh}(w') \right) . \end{aligned} \end{aligned}$$

We see that (3.2) can be rewritten as

3.8 $$\begin{aligned} \begin{aligned}&B \left( \frac{w^{\prime 2}}{ \frac{1}{k}+{\mathscr {C}}_{kh}(w') }+\frac{1}{k}+{\mathscr {C}}_{kh}(w') \right) \\&\quad -\left( \lambda +\mu -2gw \right) \left( w'^2+\left( \frac{1}{k}+{\mathscr {C}}_{kh}(w') \right) ^2\right) =0 \end{aligned} \end{aligned}$$

with $w\in C_{2\pi ,o}^{1,\alpha }, \ \mu \in {\mathbb {R}}$ and $\lambda \in {\mathbb {R}}$. By means of (3.5)-(3.6) we notice that $w=0 \in C_{2\pi ,o}^{1,\alpha }$ and $\mu =0$ is a solution of (3.8) for all $\lambda \in {\mathbb {R}}$. We now apply the Crandall–Rabinowitz theorem [34] on bifurcation from simple eigenvalues in order to prove the existence of nontrivial solutions to (3.8). To this end note that equation (3.8) can be written as ${\mathscr {F}}(\lambda ,(\mu ,w))=0$ where ${\mathscr {F}}:{\mathbb {R}} \times {\mathbb {X}} \rightarrow {\mathbb {Y}}$ is given by the expression on the left-hand side of (3.8) and the function spaces ${\mathbb {X}}$ and ${\mathbb {Y}}$ are defined as

$$\begin{aligned} {\mathbb {X}} = {\mathbb {R}} \times C^{p+1,\alpha }_{2\pi ,o,e},\quad {\mathbb {Y}} = C^{p,\alpha }_{2\pi ,e}, \end{aligned}$$

with $$C^{p,\alpha }_{2\pi ,e} = \{f \in C^{p,\alpha }_{2\pi }:f(x) = f(-x)\,\, \mathrm{for}\,\,\mathrm{all}\,\,x\in {\mathbb {R}}\}$$, $$C^{p,\alpha }_{2\pi ,o,e} = \{f \in C^{p,\alpha }_{2\pi ,o}:f(x) = f(-x)\,\, \mathrm{for}\,\,\mathrm{all}\,\, x \in {\mathbb {R}}\}$$.

Since ${\mathscr {F}}(\lambda ,(0,0))=0$, the first condition on the local bifurcation theorem is verified. We now compute the derivative of ${\mathscr {F}}$ with respect to the $(\mu , w)$ variable. Using Lemma 11, we have

3.9 $$\begin{aligned} \begin{aligned} \partial _{(\mu ,w)}{\mathscr {F}}(\lambda ,(0,0))(\theta ,l)&= -\frac{\lambda }{k} {\mathscr {C}}_{kh}(l') +\frac{gl}{k^2} - \frac{\theta }{k^2} = -\frac{1}{k^2}(\lambda k {\mathscr {C}}_{kh}(l') - gl) -\frac{\theta }{k^2}. \end{aligned} \end{aligned}$$

The expression from (3.9) can be detailed by means of Lemma 10 whose utilization allows us to infer

3.10 $$\begin{aligned} \partial _{(\mu ,w)}{\mathscr {F}}(\lambda ,(0,0))(\theta ,l) = - \frac{1}{k^2} \sum _{n=1}^{\infty } (\lambda (k n) \coth (nkh) - g) a_n \cos (nx) - \frac{\theta }{k^2} \end{aligned}$$

if $$l=\sum _{n=1}^{\infty }a_n \cos (nx)$$.

Now, using Lemma 11 it follows that the bounded linear operator $\partial _{(\mu ,w)} {\mathscr {F}}(\lambda ,(0,0)):{\mathbb {X}}\rightarrow {\mathbb {Y}}$ is invertible whenever

$$\begin{aligned} \lambda (kn) \coth (nkh) - g \ne 0 \end{aligned}$$

for any integer $n\ge 1$. Hence, in order for bifurcation to occur, it is necessary that there is at least one $n\in {\mathbb {N}}$ such that

3.11 $$\begin{aligned} \lambda (kn) \coth (nkh) - g = 0 , \end{aligned}$$

that is, the special values of $\lambda$ for which bifurcation arises are given as

$$\begin{aligned} \lambda ^{*n}=\frac{g\tanh (nkh)}{nk}. \end{aligned}$$

However, in order to make sure that the second condition in Theorem 12 is satisfied, we need to verify that $\lambda ^{*n}\ne \lambda ^{*p}$ whenever $n,p\in {\mathbb {N}}$ with $n\ne p$. The latter condition is indeed satisfied, since the function $x\rightarrow \frac{\tanh (x)}{x}$ is injective.

Since we are looking for solutions of (3.11) of minimal period $2\pi$, we can take $n=1$ in (3.11). We thus set

3.12 $$\begin{aligned} \lambda ^*:=\lambda ^{*1} = \frac{g}{k} \tanh (kh) \end{aligned}$$

to denote the solution of (3.11) corresponding to $n=1$. The previous discussion shows that $\ker (\partial _{(\mu ,w)}{\mathscr {F}}(\lambda ^*,0))$ is one-dimensional.

We are left with the examination of the transversality condition from the Crandall–Rabinowitz theorem. It is easy to see that ${\mathscr {R}}(\partial _{(\mu ,w)}{\mathscr {F}}(\lambda ^*,0))$ is the closed subspace of ${\mathbb {Y}}$ consisting of all functions $f \in {\mathbb {Y}}$ which satisfy

$$\begin{aligned} \mathop {\mathrm {\int }}\limits _{-\pi }^{\pi }f(x)\cos (x)dx=0 , \end{aligned}$$

and therefore ${\mathbb {Y}}/{\mathscr {R}}(\partial _{(\mu ,w)}{\mathscr {F}}(\lambda ^*,0))$ is the one-dimensional subspace of ${\mathbb {Y}}$ generated by the function $w^*(x) = \cos (x)$. Using (3.9) we find that

$$\begin{aligned} \partial ^2_{\lambda ,(\mu ,w)}{\mathscr {F}}(\lambda ^*,(0,0))(1,(0,w^*))=-\frac{1}{k}\lambda ^{*} {\mathscr {C}}_{kh}(w^*{}') . \end{aligned}$$

For $w^* = \cos {x}$ we have from Lemma 11 that ${\mathscr {C}}_{kh}(w^*) = \coth {(kh)} \sin x$ and ${\mathscr {C}}_{kh}(w^*{}')$ = $({\mathscr {C}}_{kh}(w^*))'$ = $\coth {(kh)} \cos x$ = $\coth (kh)w^*$, and therefore,

$$\begin{aligned} \partial ^2_{\lambda ,(\mu ,w)}{\mathscr {F}}(\lambda ^*,(0,0))(1,(0,w^*))=-\frac{1}{k} \coth (kh) w^* \notin {\mathscr {R}}(\partial _{(\mu ,w)}{\mathscr {F}}(\lambda ^*,(0,0))) \end{aligned}$$

since by (3.12) we have

$$\begin{aligned} -\frac{\lambda ^*}{k}\coth (kh)\mathop {\mathrm {\int }}\limits _{-\pi }^{\pi }w^*(x)\cos x \,dx = -\frac{\pi g}{k^2}\ne 0 . \end{aligned}$$

From the local bifurcation theorem we obtain the bifurcation values

3.13 $$\begin{aligned} \lambda = \frac{g}{k} \tanh {(kh)}, \end{aligned}$$

which lead, via (3.5), to the corresponding values for the flow force $S_0$ as

3.14 $$\begin{aligned} S_0 = \frac{g h}{k}\tanh (kh)+ \frac{gh^2}{2}. \end{aligned}$$

In light of the previous considerations, the following result on the existence of exact small amplitude periodic gravity irrotational water waves emerges.

### Theorem 7

For any $h>0,\,k>0$ and $S_{0}\in {\mathbb {R}}$ there are laminar flows with a flat free surface in water of depth h and modified flow force at the surface $S_0$. The laminar flows with modified flow force $S_0$ at the surface are exactly those with horizontal speeds at the flat free surface equal to $\sqrt{\lambda }$ given by (3.13). The values of $S_0$ of the flow force given by (3.14) trigger the appearance of periodic gravity steady waves of small amplitude, with period $\frac{2\pi }{k}$ and conformal mean depth h, which have a smooth profile with one crest and one trough per period, monotone between consecutive crests and troughs and symmetric about any crest line.

### Proof

Using the argument from the proof of Theorem 5.2 in [16] we see that $w=0$ gives rise to laminar flows in the fluid domain bounded below by the rigid bed ${\mathscr {B}}$ and above by the flat free surface $Y=h$. These laminar flows are given by

3.15 $$\begin{aligned} S(X,Y) = -\frac{g}{2}Y^2 + \left( \frac{S_0}{h} + \frac{g h}{2}\right) Y , \quad X \in {\mathbb {R}}, \,0 \le Y \le h. \end{aligned}$$

Concerning the existence of waves of small amplitude with the properties mentioned in the statement of the theorem we apply the Crandall–Rabinowitz theorem which asserts the existence of the local bifurcation curve

$$\begin{aligned} \{(\lambda (s),(0+o(s),s\cos {x} + o(s))):|s|<\varepsilon \} \subset {\mathbb {R}} \times {\mathbb {X}} \end{aligned}$$

consisting of the solutions of (3.8) with $\lambda$ given by (3.13).

Choosing $\varepsilon$ sufficiently small and using Lemma 11 we can ensure that

$$\begin{aligned} w(x) > -h\,\,\mathrm{for}\,\,\mathrm{all}\,\, x \in {\mathbb {R}}, \end{aligned}$$

and

$$\begin{aligned} \frac{1}{k} + {\mathscr {C}}_{kh}(w')(x)>0\,\,\mathrm{for}\,\,\mathrm{all}\,\, x \in {\mathbb {R}}. \end{aligned}$$

The above inequality implies that the corresponding nonflat free surface ${\mathscr {S}}$ given by (2.32) with $v=w+h$ is the graph of a smooth function, symmetric with respect to the points obtained for the values $x=n\pi ,n \in {\mathbb {Z}}$. From

$$\begin{aligned} w(x;s) = s\cos {x} + o(s)\,\,\mathrm{in}\,\, C_{2\pi }^{p+1,\alpha }, \end{aligned}$$

we have that $sw'(x;s) < 0$ for all $x \in (0,\pi ), 0<|s|<\varepsilon ,$ for $\varepsilon > 0$ sufficiently small and $p \ge 1.$ Using the evenness of $x \rightarrow w(x;s)$ we conclude the proof of the assertion about the free surface ${\mathscr {S}}$, i.e., ${\mathscr {S}}$ has one crest and one trough per minimal period and is monotone between consecutive crests and troughs. $\square$

### Remark 8

Further, we notice from (3.15) that

3.16 $$\begin{aligned} S_{Y|Y=h} = (c-u_{1})^{2}|_{Y = h}= \frac{S_0}{h} - \frac{g h}{2}=\lambda , \end{aligned}$$

that is, for laminar flows the (relative) horizontal velocity at the free surface coincides with $\sqrt{\lambda }$. Moreover, since $\lambda$ is strictly positive, we conclude from (3.16) that stagnation points occur nowhere in the bifurcation inducing laminar flows (3.15).

## Appendix

This section recalls additional properties of the operators ${\mathscr {G}}_d$ and ${\mathscr {C}}_d$, proved in [16] and used in Sect. 3, alongside with the abstract result on bifurcation from simple eigenvalues of Crandall and Rabinowitz, cf. [17].

### Lemma 9

(*i*): The operator ${\mathscr {G}}_{d}:C_{2\pi }^{p,\alpha }\rightarrow C_{2\pi }^{p-1,\alpha }$ is a bounded linear operator.
(*ii*): If the function *w* takes the constant value *c* then 3.17 $$\begin{aligned} {\mathscr {G}}_{d}(c)=\frac{c}{d}. \end{aligned}$$

### Lemma 10

Assume that

$$\begin{aligned} w=\sum _{n=1}^{\infty }a_{n}\cos (nx)+\sum _{n=1}^{\infty }b_{n}\sin (nx), \end{aligned}$$

is the Fourier series expansion of $w\in L_{2\pi ,\text {o}}^{2}$. Then

3.18 $$\begin{aligned} {\mathscr {C}}_{d}(w)=\sum _{n=1}^{\infty }a_{n}\coth (nd)\sin (nx)-\sum _{n=1}^{\infty }b_{n}\coth (nd)\cos (nx) \end{aligned}$$

### Lemma 11

(*i*): For all $d>0, \, p\in {\mathbb {N}}$ and $\alpha \in (0,1)$ it holds that ${\mathscr {C}}_d: C_{2\pi , o}^{p,\alpha }\rightarrow C_{2\pi , o}^{p,\alpha }$ is a bounded linear operator. Moreover, ${\mathscr {C}}_{d}^{-1}:C_{2\pi ,\text {o}}^{p,\alpha }\rightarrow C_{2\pi ,\text {o}}^{p,\alpha }$ is also a bounded linear operator.
(*ii*): ${\mathscr {G}}_{d}(w)=({\mathscr {C}}_{d}(w))^{\prime }={\mathscr {C}}_{d}(w^{\prime })$ for all $w\in C_{2\pi , o}^{p,\alpha }$ mit $p\ge 1$.
(*iii*): If $w\in C_{2\pi }^{p,\alpha }$ then ${\mathscr {G}}_{d}(w)=\frac{\left[ w\right] }{d}+{\mathscr {C}}_{d}(w^{\prime })$, where [*w*] denotes the average of *w* over one period.

### Theorem 12

Let ${\mathbb {X}}$ and ${\mathbb {Y}}$ be Banach spaces, I an open interval in ${\mathbb {R}}$ containing $\lambda ^*$, and ${\mathscr {F}}: I \times {\mathbb {X}} \rightarrow {\mathbb {Y}}$ be a continuous map satisfying the following properties:

1. $${\mathscr {F}}(\lambda ,0)=0\,\,\mathrm{for}\,\,\mathrm{all}\,\,\lambda \in \ I$$;
2. $\partial _\lambda {\mathscr {F}}, \, \partial _u {\mathscr {F}}\ \mathrm{and}\ \partial ^2_{\lambda ,u}{\mathscr {F}}$ exist and are continuous;
3. ${\mathscr {N}}(\partial _u {\mathscr {F}}(\lambda ^*,0))$ and ${\mathbb {Y}}/{\mathscr {R}}(\partial _u {\mathscr {F}}(\lambda ^*,0))$ are one-dimensional, with the null space generated by $u^*$;
4. $$\partial ^2_{\lambda ,u}{\mathscr {F}}(\lambda ^*,0)(1,u^*) \notin {\mathscr {R}}(\partial _u {\mathscr {F}}(\lambda ^*,0))$$.

Then there exists a continuous local bifurcation curve $\{\lambda (s), u(s):|s| < \varepsilon \}\ with\ \varepsilon >0$ sufficiently small such that $(\lambda (0),u(0)) = (\lambda ^*,0)$ and there exists a neighborhood ${\mathscr {O}}$ of $(\lambda ^*,0) \in I \times {\mathbb {X}}$ such that

$$\begin{aligned} \{(\lambda ,u) \in {\mathscr {O}} : u \ne 0, {\mathscr {F}}(\lambda ,u)=0\} = \{\lambda (s), u(s): 0< |s|<\varepsilon \}. \end{aligned}$$

Moreover, we have

$$\begin{aligned} u(s) = su^*+o(s)\ in\ {\mathbb {X}},\,\,|s|<\varepsilon . \end{aligned}$$

If $\partial _u^2{\mathscr {F}}$ is also continuous, then the curve is of class $C^1$.
